# Evaluation of polidocanol for the treatment of anal fistula: an experimental study in rats

**DOI:** 10.1590/acb416826

**Published:** 2026-11-09

**Authors:** Paulo Cesar de Castro, Janice Mery Chicarino de Oliveira Coelho, Rodrigo Rego Lins, Ruy Garcia Marques

**Affiliations:** 1 Universidade do Estado do Rio de Janeiro – Faculty of Medical Sciences – Department of General Surgery – Rio de Janeiro (RJ) – Brazil.; 2 Universidade do Estado do Rio de Janeiro – Faculty of Medical Sciences – Department of Pathology and Laboratory – Rio de Janeiro (RJ), Brazil.

**Keywords:** Rectal Fistula, Polidocanol, Models, Animal, Rats, Wistar

## Abstract

**Purpose:**

Anal fistula treatment remains challenging, with no single ideal surgical approach. This study introduces a novel technique using polidocanol, a sclerosing agent successful in varicose vein therapy, evaluating its local effects on surgically induced anal fistulas in rats.

**Methods:**

In 30 Wistar rats, two separate anal fistulas were created per animal, allowing each rat to serve simultaneously as control (saline-treated) and intervention subject (polidocanol-treated). Outcomes were evaluated at two and four weeks post-injection. Drug effects on the tract and surrounding tissues were assessed via macroscopic and microscopic examinations. Macroscopic analysis focused on local necrosis and external orifice persistence. Histopathology evaluated tract persistence, epithelial lining, and adjacent fibrosis or necrosis, while histomorphometric quantified luminal epithelial tissue.

**Results:**

Histopathology documented polidocanol’s intended primary action: destruction of the epithelial layer of the fistulous lumen, corroborated by histomorphometry. Both the mean epithelial height and the epithelial-to-lumen area ratio were significantly lower in the polidocanol-treated group.

**Conclusion:**

Local polidocanol injection effectively destroys the epithelial lining of the tract, representing a viable, minimally invasive therapeutic alternative for obliterating anal fistulas.

## Introduction

The most frequent etiology of anal fistula is the suppurative progression of an anorectal abscess, based on the cryptoglandular origin theory^1,2^. When bacteria and debris accumulate and obstruct the anal glands, an infection develops, leading to the most common formation of anorectal abscess, as proposed by Eisenhammer in the 1950s^3^.

Fistulas originating from cryptoglandular infection may possess a fully or partially epithelialized tract^4^. This may explain the recurrence of the condition when the fistulous tract is not completely eradicated during therapeutic intervention. This issue highlights the importance of the complete removal of the tract’s innermost layer to minimize the likelihood of recurrence^4,5^.

The fistulous tract perpetuates the pathological process. Therefore, the ablation or removal of this tissue segment is the primary goal of various therapeutic approaches^3,6^. The main challenge lies in eradicating the tract while minimizing damage to adjacent structures, particularly the sphincter complex.

This same principle has been successfully applied in the treatment of lower limb varicose veins. To avoid damaging structures surrounding the varicose vessels, a substance with lytic action is injected. This destroys the vessel’s endothelial layer, leading to the obliteration of its lumen and the consequent resolution of the condition^7^.

Polidocanol, originally developed as a local anesthetic, is currently one of the most widely used vascular sclerosing agents^8^. Its mechanism of action is based on its surfactant properties. Polidocanol molecules form micelles that bind to lipids in the endothelial cell membrane, dissolving essential components and causing cell lysis^7^. This endothelial damage triggers a localized fibrotic response, culminating in vascular occlusion^9^.

We hypothesized that this same principle could be applied to the treatment of anal fistulas. Therefore, an experimental study using rats was proposed to observe the effects of polidocanol on the cells of induced anal fistula tracts, as well as its potential action on adjacent tissues. Then, the objective of this study was to evaluate the action of polidocanol on the fistulous tract in an experimental model of induced anal fistula in rats.

## Methods

The study protocol was approved by the Institutional Animal Care and Use Committee of the Universidade do Estado do Rio de Janeiro (UERJ) under number 043/2023. Animal experiments complied with the Animal Research: Reporting of In Vivo Experiments (ARRIVE) guidelines. This prospective experimental study utilized Wistar rats and followed the anal fistula induction model proposed by Arakaki et al.^10^.

The experiment consisted of the following stages:

- Thirty rats underwent the induction of two anal fistulas, each using size 5 surgical steel wire and a 48-mm, 1/2 circle cutting needle. The needle was passed from the lower rectum to the lateral perianal region, approximately 1 cm from the anal verge, bilaterally. The wires were then secured as loose setons, traversing the anal sphincter complex (Fig. 1);
  **Figure 1:**
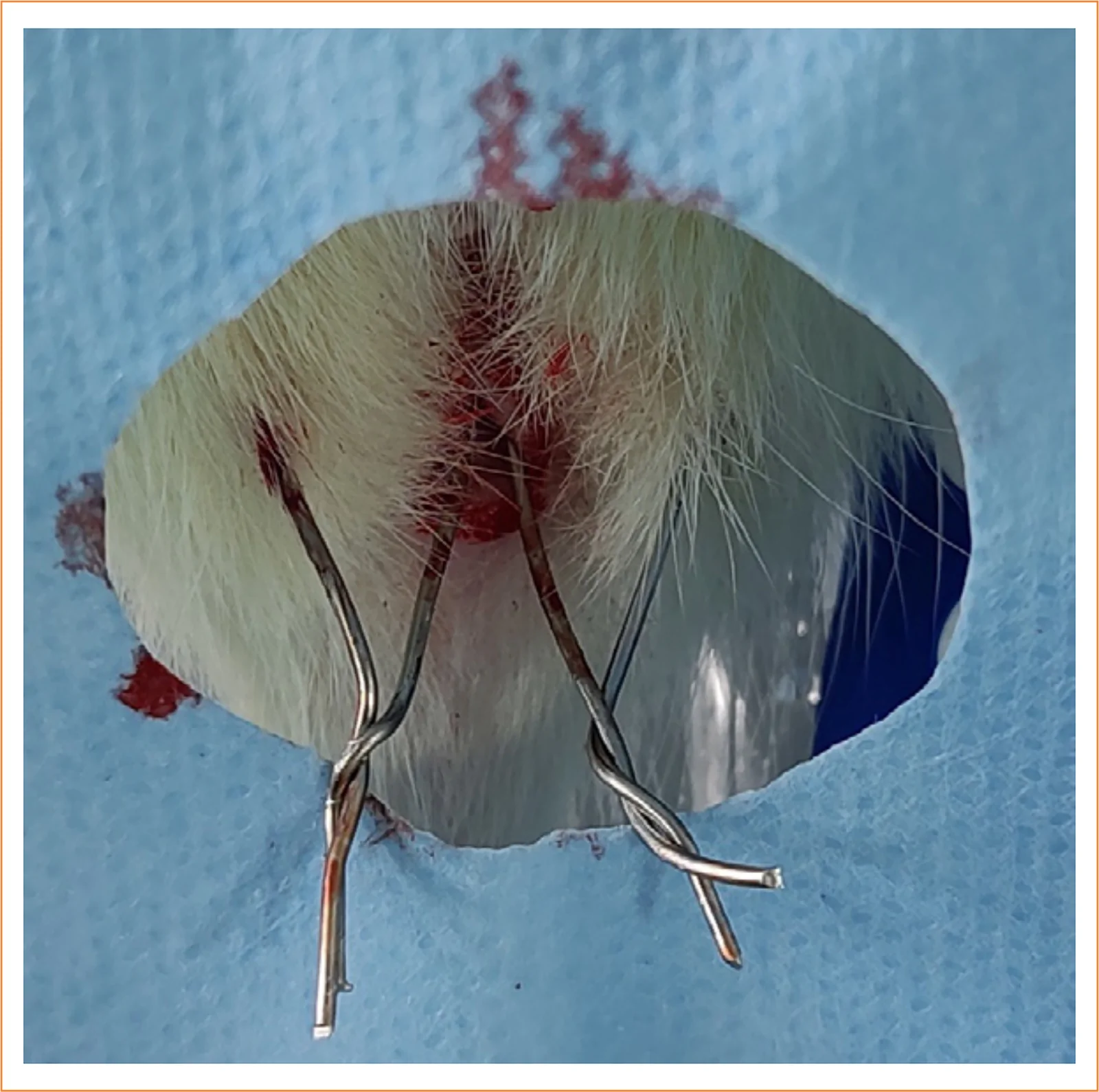
Seton placement. Setons passed bilaterally (3 and 9 o’clock).

Source: Elaborated by the authors.
- Thirty days after this initial procedure, all rats underwent a second intervention to remove the metallic setons. One of the formed fistulas, selected randomly, was filled with polidocanol foam, while the contralateral fistula was filled with 0.9% saline solution. The polidocanol foam was produced by mixing one part 3% polidocanol with four parts ambient air, as described by Tessari et al.^11^ for vessel sclerotherapy, and prepared immediately before application;
- At this stage, the animals were equally distributed into two groups:
  - In the first group (group I), animals were euthanized two weeks after the second procedure (polidocanol) to obtain tissue for histopathological and histomorphometric study;
  - The second half of the animals (group II) underwent the same process but were euthanized four weeks after the polidocanol stage.

All animals were obtained from the Animal Facility of the Laboratory of Experimental Surgery at the Faculty of Medical Sciences at UERJ and maintained under controlled temperature and lighting conditions. The rats received standard rodent chow and water *ad libitum* throughout the experimental period and were housed in collective cages, with a maximum of three animals per cage. Surgical procedures were preceded by anesthesia using a solution prepared with 0.5 mL of 2% xylazine hydrochloride, 1 mL of 10% ketamine hydrochloride, 0.6 mL of 0.5% midazolam maleate, and 8.5 mL of 0.9% saline solution. This solution was administered intraperitoneally at a volume of 1 mL per 100 g of body weight. Neither pre-anesthetic fasting nor postoperative dietary restrictions were applied.

To obtain specimens for histopathological and histomorphometric analyses, the animals were re-anesthetized as previously described and euthanized by intracardiac injection of a thiopental overdose. Following trichotomy of the perianal region and identification of the two external fistulous orifices, *en bloc* resection of the perianal area was performed, encompassing both external openings, the anus, and the lower rectum. The surgical specimen was subsequently sectioned longitudinally in the sagittal plane to allow separate evaluation of each fistulous tract. The resected tissue was then fixed in a 10% buffered formalin solution.

The anatomical specimens obtained were identified by random numbers, ensuring blinding during histopathological and histomorphometric analyses.

Histological slides were prepared from longitudinal sections of the fistula and stained with hematoxylin and eosin (H&E). This material was used for both histopathological and histomorphometric analyses.

The histopathological analysis evaluated the persistence of the fistulous tract; degree of healing; presence of fibrosis; description of cellularity and observed inflammatory pattern; presence or absence of epithelial cells in the fistula tract; and identification of granulation tissue along the fistula or tissue necrosis surrounding the fistulous tract. Any other observed characteristics were also recorded.

The histomorphometric analysis quantitatively evaluated changes in the luminal lining epithelium, the expected target of the direct action of the polidocanol foam. For this purpose, images allowing the identification of the fistula tract in a complete cross-section were used. Slides showing a longitudinal section of the fistula tract, or where the entire luminal perimeter could not be identified, or where the fistulous tract was unidentifiable were excluded from this evaluation. When more than one cross-section of the fistula was visible on the same slide, measurements were taken for each section, and the arithmetic mean of the different measurements was used as the result for that specimen. Histomorphometric measurements were performed using ImageJ software (version 1.54p, National Institutes of Health, United States of America).

The area of the fistula lumen (circumscribed by the inner edge of the luminal epithelium) and the epithelial area (between the inner and outer edges of this epithelium) were measured (Fig. 2). The mean epithelial height was obtained from the arithmetic mean of 16 equidistant measurements of epithelial thickness (Fig. 3). To ensure reproducibility, each parameter was evaluated in triplicate, and the mean of the three measurements was used as the final result. Additionally, the epithelial area/luminal area ratio was calculated as an index to normalize variability between lumen diameter and epithelial thickness.

**Figure 2:**
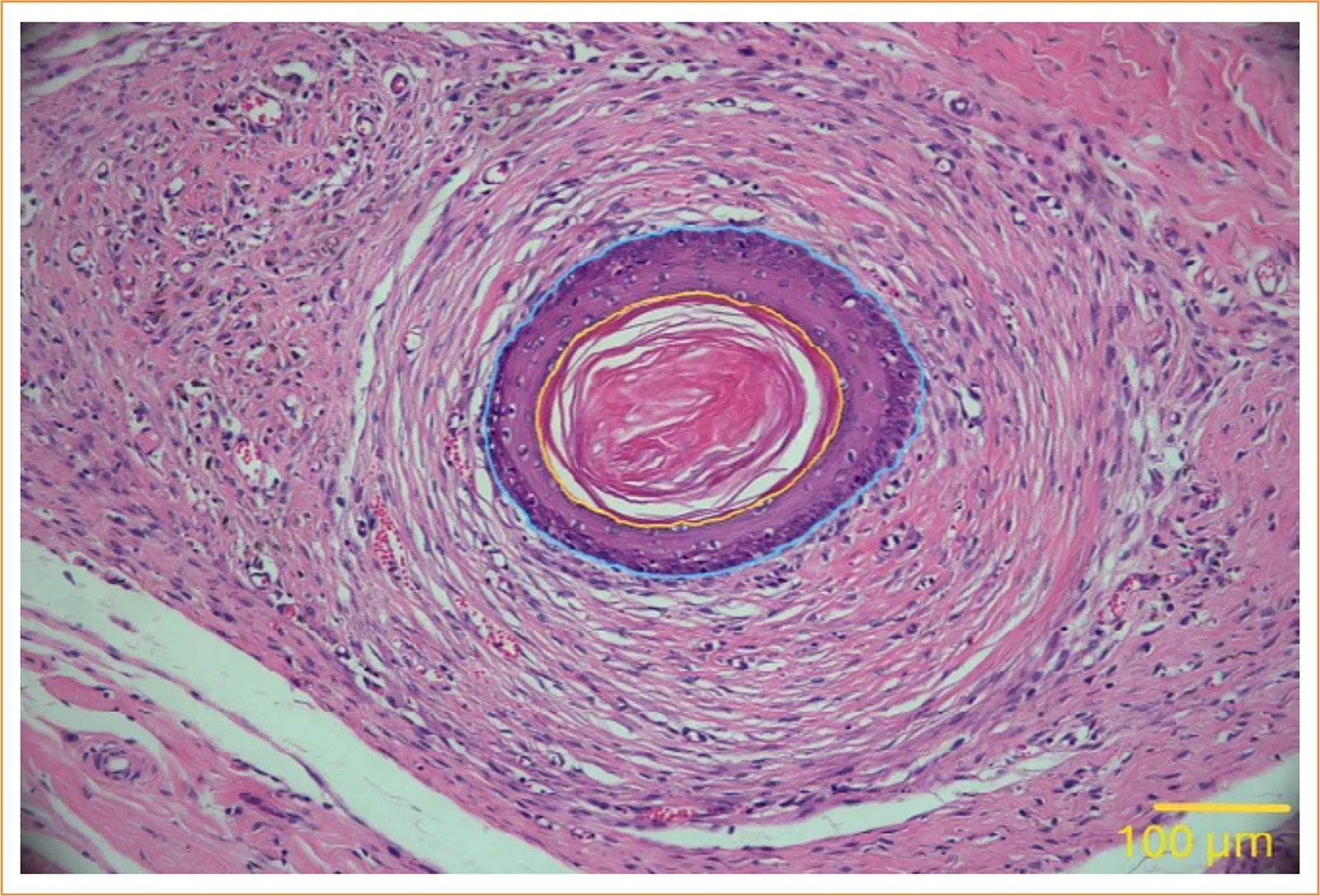
Delineation of epithelial boundaries and lumen area. Hematoxylin and eosin-stained transverse section of an anal fistula, showing the demarcation of the inner (yellow line) and outer (blue line) boundaries of the fistula epithelium and the lumen area (enclosed by the yellow line). The epithelial area is contained between the yellow and blue lines.

Source: Elaborated by the authors.

**Figure 3:**
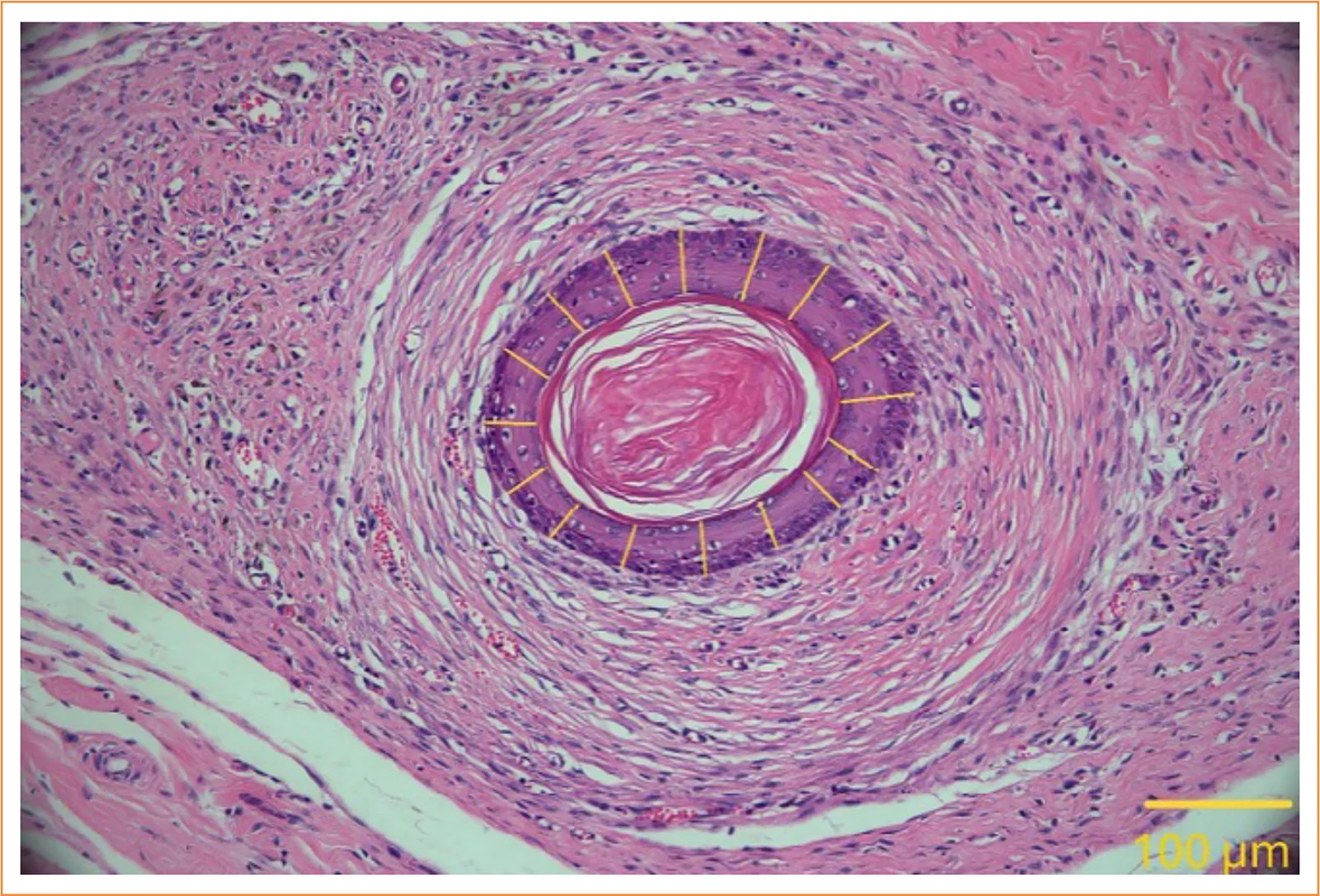
Measurement of epithelial height. Hematoxylin and eosin-stained transverse section of an anal fistula, highlighting the 16 equidistant measurements of the luminal epithelial height (yellow lines).

Source: Elaborated by the authors.

Statistical analysis employed appropriate tests, considering the type and distribution of variables. For histopathological analysis, the χ^2^ test^12^ was used to evaluate categorical variables, and the strength of association was quantified by calculating Cramér’s V coefficient^13^.

For histomorphometric analysis, the normality of data distribution for continuous variables was verified using the Shapiro-Wilk’s test^14^ and visual inspection of Q-Q plots^15^. For comparison between groups, the non-parametric Mann-Whitney’s U test^16^ was applied, and the effect size was quantified by the rank-biserial correlation coefficient (rrb)^17^. To better understand the direction of the magnitude found in rrb, the control group was conventionally coded as 0 and the intervention group as 1. Thus, a positive rrb value indicated an association trend toward the group coded as 1 (intervention group).

## Results

The study began with 30 Wistar rats (initial median weight = 298.5 g; interquartile range [IQR] = 270–366). At the conclusion, the median weight was 388 g (IQR = 369–412), with a median daily weight gain of 1.65 g/day (IQR = 0.94–1.87). A sample success rate of 96.7% (29/30 animals) was achieved, with one animal excluded due to bilateral seton loss. Six animals with unilateral loss were retained in the intervention group. A total of eight setons were lost, leaving 52 fistulas for final analysis (23 in the control group and 29 in the intervention group). No mortality was recorded.

All remaining 52 fistulas were identified by macroscopic observation of the external orifice scar at two and four weeks following metallic wire removal. Four fistulas (7.7%) exhibited external signs of local infection, characterized by undrained abscess formation. All four abscesses occurred on the polidocanol intervention side, with one observed in the two-week evaluation group and three in the four-week evaluation group.

In the histopathological analysis, results from the control and intervention groups were compared without considering the time factor. The presence of epithelial cells within the fistulous tract, when identifiable, was lower in the polidocanol-treated group [χ^2^ (4, N = 27) = 7.53, *p* = 0.110, V = 0.53], although the rate of tract identification was similar between groups [χ^2^ (2, N = 52) = 0.00, *p* = 1.000, V = 0.00]. Adverse effects associated with polidocanol, such as an increased inflammatory response, necrosis, and abscess formation, showed no statistically significant differences between groups (Table 1).

**Table 1:** Results of histopathological analysis, comparing control and intervention groups.

Control = 23 Intervention = 29 Response – Yes [n]	Control [23]	Intervention [29]	*p* -value	V
**Internal opening or thread identified? (%)**	15 (65)	23 (79)	1.000	0.00
**External opening identified? (%)**	9 (39)	16 (55)	0.384	0.12
**Fistulous tract or remnant thereof identified on microscopy? (%)**	11 (48)	16 (55)	1.000	0.00
**Presence of fistulous tract? (%)**			0.656	0.13
Complete	9 (39)	11 (38)		
Partially free	2 (9)	5 (17)		
Undetectable	12 (52)	13 (45)		
**Presence of epithelial cells in the fistula tract? Excluding ‘undetectable’ (item 4)**	[11]	[16]	0.110	0.53
Absence	1 (9)	5 (31)		
Entire tract	8 (73)	4 (25)		
Parts of the tract < 10% (mild)	0 (0)	2 (13)		
Parts of the tract between 20 and 80% (moderate)	0 (0)	2 (13)		
Parts of the tract > 90% (abundant)	2 (18)	3 (19)		
**Presence of granuloma in the fistula tract? (%)**			0.218	0.33
Absence	3 (13)	6 (21)		
Entire tract	2 (9)	0 (0)		
Parts of the tract < 10% (mild)	14 (61)	13 (45)		
Parts of the tract between 20 and 80% (moderate)	4 (17)	8 (28)		
Parts of the tract > 90% (abundant)	0 (0)	2 (7)		
Inflammatory response pattern* (%)			0.381	0.19
Diffuse	3 (13)	5 (17)		
Granulomatous	20 (87)	22 (76)		
Both	0 (0)	2 (7)		
Presence of giant cells?* (%)			0.277	0.22
Absence	3 (13)	5 (17)		
Foreign body type cells	7 (30)	14 (48)		
Foreign body and Langhans type cells	13 (57)	10 (34)		
**Presence of fibrosis? (%)**			0.303	0.27
Absence	9 (39)	9 (31)		
< 10% (mild)	7 (30)	14 (48)		
Between 20 and 80% (moderate)	5 (22)	2 (7)		
> 90%	2 (9)	4 (14)		
**Presence of signs of tissue necrosis in the specimen? (%)**		0.229	0.24	1-2 mg/kg
Absence	20 (87)	20 (69)		
Near the tract	3 (13)	7 (24)		
Distant from the fistulous tract	0 (0)	2 (7)		
**Abscess (%)**	1 (4)	7(24)	0.115	0.22

* May have more than one response;

*p*-value: χ^2^ test; V: Cramér’s V coefficient.

Source: Elaborated by the authors.

Subsequently, considering the time factor, results from the groups were evaluated at two and four weeks. Although histopathological variables did not demonstrate statistically significant differences between groups across time points (Table 2), notable trends were observed. Specifically, both the presence of epithelial cells within the tract [χ^2^ (4, N = 16) = 3.53, *p* = 0.473, V = 0.47] and the presence of fibrosis [χ^2^ (3, N = 29) = 7.23, *p* = 0.065, V = 0.50] exhibited moderate-to-strong association coefficients (Cramér’s V) despite p-values above the significance threshold.

**Table 2:** Results of the histopathological analysis, considering the time factor (group I and group II).

Groups I and II separated Control (c) / Intervention (i) Response – Yes [n]	Group I			Group II		*p* -value	V
	c	i		c	i		
	[11]	[15]		[12]	[14]		
**Internal opening or thread identified? (%)**	7 (64)	14 (93)		8 (67)	9 (64)	0.330	0.18
**External opening identified? (%)**	4 (36)	9 (60)		5 (42)	7 (50)	1.000	0.00
**Fistulous tract or remnant thereof identified on microscopy? (%)**	5 (45)	8 (53)		6 (50)	8 (57)	1.000	0.00
**Presence of fistulous tract? (%)**						0.846	0.11
Complete	3 (27)	6 (40)		6 (50)	5 (36)		
Partially free	2 (18)	2 (13)		0 (0)	3 (21)		
Undetectable	6 (55)	7 (47)		6 (50)	6 (43)		
**Presence of epithelial cells in the fistula tract? (%) Ŧ**	[5]	[8]		[6]	[8]	0.473	0.47
Absence	1 (20)	2 (25)		0 (0)	3 (38)		
Entire tract	3 (60)	3 (38)		5 (83)	1 (13)		
Parts of the tract < 10% (mild)	0 (0)	1 (13)		0 (0)	1 (13)		
Parts of the tract between 20 and 80%	0 (0)	0 (0)		0 (0)	2 (25)		
Parts of the tract > 90% (abundant)	1 (20)	2 (25)		1 (17)	1 (13)		
**Presence of granuloma in the fistula tract? (%)**					0.367	0.33	
Absence	1 (9)	3 (20)		2 (17)	3 (21)		
Entire tract	0 (0)	0 (0)		2 (17)	0 (0)		
Parts of the tract < 10% (mild)	8 (73)	5 (33)		6 (50)	8 (57)		
Parts of the tract between 20 and 80%	2 (18)	5 (33)		2 (17)	3 (21)		
Parts of the tract > 90% (abundant)	0 (0)	2 (13)		0 (0)	0 (0)		
Inflammatory response pattern* (%)						0.309	0.29
Diffuse	1 (9)	3 (20)		2 (17)	2 (14)		
Granulomatous	10(91)	12(80)		10(83)	10(71)		
Both	0 (0)	0 (0)		0 (0)	2 (14)		
Presence of giant cells?* (%)						0.233	0.32
Absence	1 (9)	3 (20)		2 (17)	2 (14)		
Foreign body type cells	6 (55)	5 (33)		1 (8)	9 (64)		
Foreign body and Langhans type cells	4 (36)	7 (47)		9 (75)	3 (21)		
**Presence of fibrosis? (%)**						0.065	0.50
Absence	3 (27)	4 (27)		6 (50)	5 (36)		
< 10% (mild)	4 (36)	5 (33)		3 (25)	9 (64)		
Between 20 and 80% (moderate)	3 (27)	2 (13)		2 (17)	0 (0)		
> 90%	1 (9)	4 (27)		1 (8)	0 (0)		
**Presence of signs of tissue necrosis in the specimen? (%)**				0.233	0.32		
Absence	11(100)	12(80)		9 (75)	8 (57)		
Near the tract	0 (0)	3 (20)		3 (25)	4 (29)		
Distant from the fistulous tract	0 (0)	0 (0)		0 (0)	2 (14)		
**Abscess (%)**	1 (9)	2 (13)		0 (0)	5 (36)	0.330	0.18

* May have more than one response;

p-value: χ2 test; V: Cramér’s V coefficient; Ŧ: Excluding ‘undetectable’ (item 4).

Source: Elaborated by the authors.

Measurements obtained from the histomorphometric analysis are summarized in Tables 3 and 4. A total of nine fistulas from the control group and 11 from the intervention group were included. Fistula lumen area exhibited wide variability across extremes of size, but statistical analysis revealed no significant difference between the control and intervention groups.

**Table 3:** Statistical analysis of the histomorphometric variables of the control and intervention groups<tfn href="tfn03">*</tfn>.

Variable	Control (n = 9)	Intervention (n = 11)	*p* -value	rrb
Lumen area (µm^2^)	98,077.75	1,128,242.00	0.058	0.515
	[23,491.77–386,106.23]	[175,059.47–1,354,613.80]		
Epithelium area (µm^2^)	69,886.68	14,507.12	0.092	-0.455
	[51,045.09–137,885.81]	[0–84,191.59]		
Epithelium height (µm)	43.04	12.52	0.032	-0.576
	[39.10–54.62]	[0–40.64]		
Epithelium/lumen ratio**	1.18	0.07	0.014	-0.657
	[0.33–2.17]	[0–0.27]		

* Data are presented as median and [interquartile range Q1–Q3];

** ratio of epithelium area to lumen area (dimensionless);

p-value: Mann-Whitney’s U test; rrb: rank-biserial correlation coefficient.

Source: Elaborated by the authors.

**Table 4:** Statistical analysis of the histomorphometric variables of the two- and four-week intervention groups<tfn href="tfn05">*</tfn>.

Variable	Intervention I (n = 5)	Intervention II (n = 6)	*p* -value	rrb
Lumen area (µm^2^)	149,481.24	1,160,771.79	0.429	0.333
	[23,579.04–1,452,920.27]	[519,687.38–1,240,555.90]		
Epithelium area (µm^2^)	38,484.72	7,253.56	0.924	-0.067
	[0–51,786.67]	[0–91,074.16]		
Epithelium height (µm)	28.42	6.26	0.632	-0.200
	[0–52.86]	[0–19.52]		
Epithelium/lumen ratio**	0.11	0.04	0.389	-0.333
	[0–2.20]	[0–0.10]		

Intervention I: intervention group two weeks; intervention II: intervention group four weeks;

* data are presented as median and [interquartile range Q1–Q3];

** ratio of epithelium area to lumen area (dimensionless); p-value: Mann-Whitney’s U test; rrb: rank-biserial correlation coefficient.

Source: Elaborated by the authors.

In contrast, mean epithelial height was significantly lower in the intervention group compared to controls (median = 12.52, IQR = 0.00–40.64 *versus* median = 43.04, IQR = 39.10–54.62; U = 78.0, *p* = 0.032, rrb = -0.576), reflecting a large effect size favoring reduction in the treated animals.

Similarly, the epithelial-to-lumen area ratio was significantly reduced in the intervention group compared to the control group (median = 0.27, IQR = 0.00–0.27 *versus* median = 1.18, IQR = 0.33–2.17; U = 82.0, *p* = 0.014, rrb = -0.657), also demonstrating a large negative effect size.

## Discussion

Two-time intervals were established in this study: a 30-day period between seton placement and removal, and two- and four-week intervals to evaluate the effects of polidocanol. The 30-day period followed established experimental models in rats to allow for fistula tract maturation and chronification^10^,^18–21^. The subsequent intervals for histological analysis were empirically determined, as no previous studies were found regarding the use of polidocanol foam in anal fistulas.

Polidocanol is a sclerosing agent widely used in the treatment of lower limb varicose veins. It is a synthetic surfactant with amphiphilic properties. Upon injection, the hydrophobic moiety of the molecule interacts with the lipid bilayer of the venous endothelial cell membrane^7^. This interaction disrupts membrane integrity, increasing permeability and ultimately leading to cell lysis and subsequent necrosis^7,9,22^. Research suggests that higher concentrations of polidocanol may lead to a slight increase in vessel wall damage, although this is not always statistically significant^23^. Clinical trials have evaluated the safety and efficacy of various concentrations, including 0.5, 1, 2, and 3%^7,9,22^.

The underlying rationale for the use of polidocanol in anal fistulas is its capacity to induce controlled tissue damage to the vascular endothelium^7-9^. Upon reaching the epithelial lining of the fistula tract, the agent is intended to promote epithelial denudation, thereby facilitating tissue regeneration and subsequent healing. This mechanism is designed to achieve collapse and permanent obliteration of the lumen, effectively transposing the established principles of chemical sclerotherapy from phlebology to the field of coloproctology.

Although qualitative histological assessments may produce observer bias and introduce subjectivity^10^, they are a fundamental step for subsequent semi-quantitative and quantitative evaluations. In this study, specific histopathological findings were pivotal in demonstrating the effect of polidocanol foam on the fistula tract. The most anticipated outcome of polidocanol’s action, the destruction of the tract’s internal lining, is considered a critical step toward the healing of anal fistulas^3,6^. Our samples revealed alterations that corroborate this intended action of polidocanol.

Histopathological analysis showed that the ability to identify the fistulous tract was similar between the control and intervention groups, as demonstrated by the failure to reject the null hypothesis. Conversely, the qualitative screening for epithelial cells suggested a positive correlation for the action of polidocanol, as indicated by Cramér’s V coefficient, despite not reaching statistical significance. This trend was subsequently confirmed by quantitative histomorphometric evaluation, which objectively validated the drug’s effect on the epithelial lining.

Potential adverse effects may be attributed to polidocanol. Its lytic action could theoretically lead to necrosis beyond the target area or trigger abscess formation due to the accumulation of cellular debris. In the present study, although histopathological analysis revealed a higher absolute frequency of necrosis and abscess in the intervention group, these differences did not reach statistical significance. Therefore, our data suggest that polidocanol did not increase the risk of lytic lesions beyond the luminal lining epithelium. Consequently, within this experimental model, our findings suggest that polidocanol is a safe option.

Histomorphometric analysis, in contrast to histopathological analysis, consisted of purely quantitative measurement. It directly sought to evaluate the expected local effect of polidocanol foam on the luminal epithelial layer of anal fistulas. This assessment was performed by measuring and comparing specific parameters found in the histological slides of the anal fistulas. The analysis followed the same blinding protocol used in the histopathological study.

The variables measured (lumen area, epithelial area, and mean epithelial height) required a certain degree of homogeneity for result comparison. For this analysis, only histological sections showing the fistula in its entirety and in cross-section to its axis, all within a single microscope field of view, were selected. This restriction was essential to avoid measurement bias, since a tangential or oblique cut relative to the fistula’s longitudinal axis results in artifacts, such as artifactual thickening of the epithelial layer or an illusory increase in luminal area.

In cases where a single slide contained multiple cross-sections of the same fistula, measurements were performed on all images individually. Subsequently, the arithmetic mean of these values was calculated to obtain a single representative value per fistula for the final histomorphometric analysis.

Histomorphometric analysis demonstrated significant differences between the control and intervention groups in two of the assessed variables. The intervention group exhibited a significantly lower mean epithelial height and a reduced epithelium-to-lumen area ratio. In both cases, the rrb indicated a large effect size; notably, five fistulas (45.5%) in the intervention group presented no identifiable epithelium. Regarding the fistula lumen area, which showed wide variability, the difference did not reach statistical significance. However, the large effect size (rrb) suggests a trend toward larger values in the intervention group. Taken together, these findings indicate that the intervention resulted in significant disruption of the luminal epithelium.

Consequently, histomorphometric analysis successfully demonstrated the primary expected action of polidocanol foam on the epithelium lining the anal fistula lumen. Reductions in epithelial area, mean epithelial height, and the epithelium-to-lumen area ratio were observed, all featuring moderate-to-large effect size correlation coefficients favoring the intervention group. These histomorphometric findings provided the objective evidence that complemented the qualitative trends initially observed in the histopathological analysis, confirming the drug’s efficacy in this experimental model.

To assess the temporal progression of polidocanol’s effects, histological collection was performed at two and four-week intervals to monitor the progression or regression of tissue changes. In this intertemporal comparison, no statistically significant differences were observed, demonstrating consistency in both histopathological and histomorphometric findings. This stability suggests that the sclerosing effect was maintained throughout the study period.

Investigating evidence of fistula persistence or cure is a compelling prospect. Although this study successfully documented the action of polidocanol on the anal fistula tract, its primary objective, certain considerations must be addressed. A significant limitation concerns the study’s capacity to evaluate the definitive healing of anal fistulas following polidocanol foam application. While this objective was not the original scope of our investigation, the pursuit of such evidence remains an appealing avenue for research.

Histopathological evaluation of samples provides valuable insights into cellular arrangement, tissue types, and their relationships. However, obtaining this material requires tissue harvesting, which irreversibly halts the dynamic processes of healing, definitive resolution, or potential recurrence. Consequently, this prevents the observation of medium and long-term outcomes of the treated fistulas.

Magnetic resonance imaging (MRI) offers an alternative for longitudinal monitoring, as it allows for the assessment of tract persistence and patency^24^. Furthermore, MRI enables the diagnosis and evolutionary tracking of complications, such as abscesses or fluid collections^24^. Nevertheless, performing MRI in small animals like rats requires specialized equipment that is not frequently accessible. Pigs, due to their larger size, represent a promising alternative for studies involving MRI follow-up. Moreover, they may serve as a robust experimental model for the translational phase, acting as a possible bridge to human clinical trials.

## Conclusion

The present study demonstrated the action of polidocanol foam on the fistulous tract in an induced experimental model in rats. Its primary action, which is the destruction of the epithelial layer lining the fistula lumen, was documented by histopathological analysis and substantiated by histomorphometric analysis, revealing a reduction in the thickness of this epithelium and, in some cases, its complete removal.

The lytic effects of polidocanol, however, did not cause undesirable adverse effects, such as necrosis and abscess formation, in the tissues surrounding the induced anal fistula.

## Data Availability

The datasets used and analyzed during the current study are available from the corresponding author on reasonable request.

## References

[1] Eisenhammer S (1956). The internal anal sphincter and the anorectal abscess. Surg Gynecol Obstet.

[2] Parks AG (1961). Pathogenesis and treatment of fistula-in-ano. Br Med J.

[3] Johnson EK, Bernier G, Steele SR, Hull TL, Hyman N, Maykel JA, Read TE, Whitlow CB (2022). The ASCRS textbook of colon and rectal surgery.

[4] van Koperen PJ, ten Kate FJW, Bemelman WA, Slors JFM (2010). Histological identification of epithelium in perianal fistulae: a prospective study. Colorectal Dis.

[5] Lunniss PJ, Faris B, Rees HC, Heard S, Phillips RKS (1993). Histological and microbiological assessment of the role of microorganisms in chronic anal fistula. Br J Surg.

[6] Corman ML, Corman ML (2005). Colon and rectal surgery.

[7] Gallo G, Pietroletti R, Novelli E, Sturiale A, Tutino R, Lobascio P, Laforgia R, Moggia E, Pozzo M, Roveroni M, Bianco V, Realis Luc A, Giuliani A, Diaco E, Naldini G, Trompetto M, Perinotti R, Sammarco G (2022). A multicentre, open label, single arm phase II trial of the efficacy and safety of sclerotherapy using 3% polidocanol foam to treat second degree haemorrhoids (SCLEROFOAM). Tech Coloproctol.

[8] Bastos FR, Lima AE, Assumpção AC (2009). Ecoescleroterapia de varizes com espuma: revisão de literatura. Rev Med Minas Gerais.

[9] Parsi K, Exner T, Connor DE, Herbert A, Ma DDF, Joseph JE (2008). The lytic effects of detergent sclerosants on erythrocytes, platelets, endothelial cells and microparticles are attenuated by albumin and other plasma components in vitro. Eur J Vasc Endovasc Surg.

[10] Arakaki MS, Santos CHM, Falcão GR, Cassino PC, Nakamura RK, Gomes NF, Santos RGC (2013). Experimental model of anal fistula in rats. J Coloproctol.

[11] Tessari L, Cavezzi A, Frullini A (2001). Preliminary experience with a new sclerosing foam in the treatment of varicose veins. Dermatol Surg.

[12] Pearson K. X. (1900). On the criterion that a given system of deviations from the probable in the case of a correlated system of variables is such that it can be reasonably supposed to have arisen from random sampling. Lond Edinb Dublin Philos Mag J Sci.

[13] Cramér H. (1999). Mathematical methods of statistics.

[14] Shapiro SS, Wilk MB (1965). An analysis of variance test for normality (complete samples). Biometrika.

[15] Torman VBL, Coster R, Riboldi J (2012). Normalidade de variáveis: métodos de verificação e comparação de alguns testes não-paramétricos por simulação. Rev HCPA.

[16] Mann HB, Whitney DR (1947). On a test of whether one of two random variables is stochastically larger than the other. Ann Math Stat.

[17] Cureton EE (1956). Rank-biserial correlation. Psychometrika.

[18] Galvão MO, Santos CHM, Falcão GR (2016). Evaluation of the inflammatory response induced by different materials in the treatment of perianal fistulas: experimental study in rats. J Coloproctol.

[19] Romero GT, Santos CHM, Cassino PC, Odashiro M, Seno GZ, Aléssio GF (2016). Treatment of fistula-in-ano with cyanoacrylate glue with and without prior seton placement in rats. Acta Cir Bras.

[20] Tanus OAV, Santos CHM, Dourado DM, Conde AL, Giuncanse F, Souza IF, Costa IO, Costa RL (2019). Primary sphincteroplasty comparing two different stitches in anal fistula treatment: experimental study in rats. Arq Bras Cir Dig.

[21] Coskun M, Canturk AO, Cakiroglu H, Budak O (2025). Therapeutic potential of epidermal growth factor for perianal fistulas: an experimental rat model. Updates Surg.

[22] Zheng X, Wei Q, Zhang H. (2018). Novel developments in polidocanol sclerotherapy: a review. J Biosci Med.

[23] Yılmaz S, Şahin AF, Kızıltan F, Mermi EÜ, Aksoy E, Barış Ö (2017). Early histopathological effects of fluid versus foam sclerotherapy on great saphenous vein endothelium. Turk J Vasc Surg.

[24] Dawes AJ, Lightner AL (2025). Perianal fistulizing Crohn’s disease: outcomes of surgical repairs and current state of stem cell-based therapies. Clin Colon Rectal Surg.

